# Variation of psychological and anthropometrics measures before and after dieting and factors associated with body dissatisfaction and quality of life in a Lebanese clinical sample

**DOI:** 10.1186/s40359-021-00691-7

**Published:** 2021-12-01

**Authors:** Chadia Haddad, Maha Zakhour, Hala Sacre, Nicole Eid, Georgie Wehbé, Joelle Farha, Jocelyne Azar, Sahar Obeid, Souheil Hallit

**Affiliations:** 1 Research Department, Psychiatric Hospital of the Cross, P.O. Box 60096, Jal Eddib, Lebanon; 2 INSERM, Univ. Limoges, IRD, U1094 Tropical Neuroepidemiology, Institute of Epidemiology and Tropical Neurology, GEIST, Limoges, France; 3 INSPECT-LB (Institut National de Santé Publique, d’Épidémiologie Clinique Et de Toxicologie-Liban), Beirut, Lebanon; 4 Faculty of Science, Lebanese University, Fanar, Lebanon; 5 Nutriform Clinic, Jal Eddib, Lebanon; 6 Department of Nutrition and Dietetics, Faculty of Pharmacy, Saint Joseph University (USJ), Beirut, Lebanon; 7 Army Healthcare, Lebanese Army, Beirut, Lebanon; 8 Faculty of Medicine, Lebanese American University, Byblos, Lebanon; 9 Faculty of Arts and Sciences, Holy Spirit University (USEK), Jounieh, Lebanon; 10 Faculty of Medicine and Medical Sciences, Holy Spirit University of Kaslik (USEK), Jounieh, Lebanon

**Keywords:** Weight management, Diet, Body dissatisfaction, Diet clinic, Psychological factors

## Abstract

**Objective:**

The primary objective of this study was to assess a change in the psychological states (stress, self-esteem, anxiety and depression), anthropometric measurements and physical/mental quality of life before and after diet in a sample of Lebanese subjects visiting a diet clinic. The secondary objectives included the evaluation of factors associated with body dissatisfaction, mental and physical quality of life (QOL) before the intervention of the diet program and the change in quality of life after this intervention among those participants.

**Methods:**

This cross-sectional study, conducted between May and August 2018, enrolled 62 participants recruited from three diet clinics. The QOL was measured using the 12-item Short Form Health Survey (SF-12) and the psychological states was measured using the following scales: The Rosenberg Self-esteem Scale, Perceived Stress Scale, Hamilton Anxiety Rating Scale and Hamilton Depression Rating Scale.

**Results:**

A significant reduction in body dissatisfaction, anxiety, waist, weight and body fat and a significant increase in the physical and mental quality of life was seen after diet compared to before it (*p* < 0.001 for all). No significant variation in perceived stress (*p* = 0.072), self-esteem (*p* = 0.885), and depression (*p* = 0.353) after diet were found. Higher BMI (β = 0.440) and higher anxiety (β = 0.132) were associated with higher body dissatisfaction scores, whereas higher self-esteem (β = − 0.818) was significantly associated with lower body dissatisfaction. Higher perceived stress (β = − 0.711), higher body dissatisfaction (β = − 0.480) and being a female (β = − 4.094) were associated with lower mental QOL. Higher Physical Activity Index was significantly associated with higher mental and physical QOL (β = 0.086 and β = 0.123 respectively).

**Conclusion:**

The results indicate the effectiveness of diet programs in enhancing the quality of life, psychological and anthropometric measures.

## Introduction

Over the past few years, excessive dieting has become a concern among the general population due to increased weight and obesity, which are now a global public health concern [1]. According to the World Health Organization, in 2016, more than 1.9 billion adults worldwide were overweight, of which over 650 million were obese [2]. The prevalence of weight loss and diet vary widely across studies, ranging from 9.5 [3] to 73.8% [4], while the overall prevalence of weight control attempts ranges between 37 [5] and 81.5% [6]. A recent systematic review and meta-analysis showed that 42% and 23% of adults in the general population reported trying to lose weight and maintaining weight, respectively, at some point in time [7]. Dieting has become a common aspect among people to achieve their desired body weight. The main motive for dieting is the continuous pursuit of improved health through limiting caloric intake and, consequently, weight gain and obesity. Previous findings showed that a twelve-week intervention decreased body fat percentage in the intervention group, with no significant change in weight and BMI [8]. Other studies showed that six-month interventions decreased both the BMI and the body fat percentage, the latter decreasing more quickly [9]. Improvement in appearance, energy level, self-esteem and prevention of disease were the strongest motivations for weight loss program. Health and medical concerns motivate adults to follow weight loss programs while social appearance and media pressure trigger young adults [10].

Diet and body weight can affect the mental health of the individual. Previous studies showed that dietary interventions improved body image, body size dissatisfaction, body shape concern, self-esteem and depression and, in turn, these improvements can increase the likelihood of sustaining successful weight loss [11]. These interventions are beneficial for individuals; they encourage them to follow appropriate eating habits and healthy diet and so to a self-acceptance and health attitudes [11]. Therefore, strategies that focus on reducing weight and maintaining weight loss might improve psychological outcomes. The association between mood disorders and diet remains controversial where some studies found no overall effect of diet on anxiety [12], while other research suggested positive effects of dietary interventions on subclinical depression and anxiety [13]. Similarly, self-esteem and low self-confidence, strongly related to the desire of being thinner, considerably improved after weight loss [14].

On another hand, unhealthy eating behaviors and strict dieting are associated with negative body image, body dissatisfaction (BD), affect people’s psychological well-being and can harm their physical appearance, thus leading to fatal outcomes [15]. Body dissatisfaction is defined by the negative perceptions and feelings about one’s body, has been recognized as a psychological correlate of obesity linked to disordered eating, poor self-esteem, depression, and stress [15].

From another perspective, the desire and pressure to lose weight, favored by media exposure, may lead to unhealthy weight control behaviors such as fasting, skipping meals, taking laxatives or diet pills, and food restriction. Media can play an influential role in promoting slimness as an illustration of a beautiful body and a standardized image for attractiveness. Indeed, repeated exposure to media content leads viewers to start accepting media images as a reflection of reality. However, since media presentation of women’s bodies showcases an ideal, which is out of reach to most, it may lead to a decreased satisfaction with one’s body and to behaviors aimed at meeting this ideal such as dieting, bingeing and purging and skipping meals. Equally, some studies support the fact that family and media pressures have an influence on one’s appearance, thereby creating body dissatisfaction and disturbed eating behaviors [16].

Psychological distress factors can be either associated with physical and mental quality of life and/or to BD [17]. Moreover, even though BD is not the only consequence of weight gain and fat deposit, it is also identified in people with normal weight [18]. Overweight people have negative perceptions and feelings toward their body, resulting in high levels of BD. Some studies have shown that BD is associated with impairments in various aspects of quality of life (QOL) a multidimensional concept defined as an individual’s subjective evaluation of both positive and negative aspects of one’s life [19, 20]. Other relevant outcome measures of poor QOL include stress, anxiety, depression, decreased energy, impaired concentration, social isolation, and BD [21, 22].Perceived stress may affect negatively the psychological and physiological health and decrease the quality of life [23]. Levinson et al. [24, 25] found that an increase of social anxiety was positively related to a negative body image evaluation while other evidence suggested that body dissatisfaction was positively correlated with social anxiety and self-consciousness [26, 27]. A research conducted among the Mediterranean adult population found that overweight adults were more likely to underestimate their body weight and were dissatisfied with their body image compared to those with a normal weight [28]. A rise in BMI leads to a negative attitude about one's body and appearance, overweight and obese people are more likely to have negative body image issues, and worse psychological effects, which may contribute to weight loss attempts or unhealthy habits [29, 30]. Body image education increase self-esteem and reduce body dissatisfaction [31]. Likely, physical activity (PA) has a positive effect on body image and is associated with body image improvement [32].

**Fig. 1:**
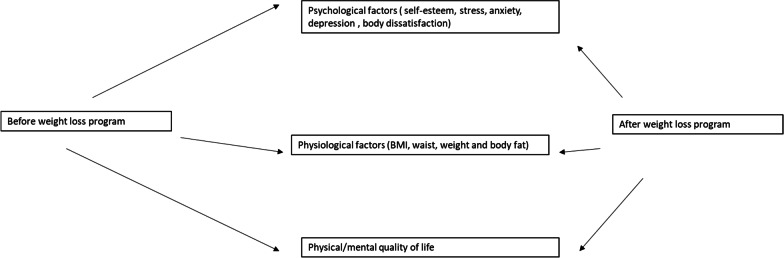
Theoretical framework of the impact of weight loss program on psychological factors physiological factors and quality of life

Little research has investigated the relationship between psychological distress, BD, and QOL. To date, studies exploring the effectiveness of weight-loss interventions targeted at specific populations did not consider mental improvement as the primary outcome. A closer look at the literature shows that several questions about the association between psychological distress and BD and QOL remain to be addressed. Therefore, the primary objective of this study was to assess changes in the psychological states (stress, self-esteem, anxiety, and depression), anthropometric measurements, and physical/mental quality of life before and after dieting in a sample of people visiting diet clinics in Lebanon (Fig. 1). Secondary objectives included the evaluation of factors associated with BD and QOL before the intervention of the diet program and the change in quality of life after this intervention among those participants.

## Methods

### Participants

This is a repeated cross-sectional study was conducted in two time intervals between May and August 2018 and enrolled 62 participants recruited from three diet clinics in Lebanon (two in Mount Lebanon and one in Beirut). The patients were first time enrolled in May 2018 and another assessment for the same participants was done after 12 week on August 2018.

Eligible participants were all healthy individuals 18 years and older, newly consulting for weight loss at a diet clinic. Those with concurrent disease or clinical psychopathology, known to alter weight, were excluded. For the purpose of this study, all three clinics adopted the same 12-week weight loss program. Before enrolling, the head dietitian briefed participants on the study objectives and methodology and assured them of the anonymity of their participation. They had the right to accept or refuse to participate, and no financial compensation was offered in exchange for their participation. The main motivation of the participants for weight loss was improvements in appearance by lowering their weight, self‐confidence and energy level.

### Sample size calculation

**Fig. 2:**
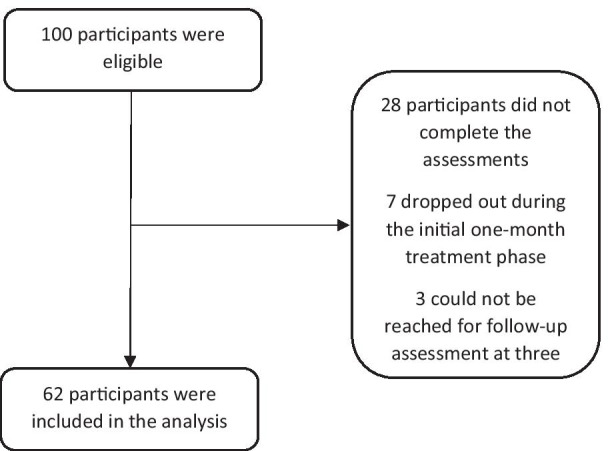
Enrollment of participants

The G-Power software version 3.1.9.2 was used to calculate the minimum sample size for this study, with a 1 − β = 0.8 and an effect size of 0.55, based on the mean ± SD of Body Mass Index (BMI) in a sample of obese individuals attending weight management clinic [33]. The minimum sample required was 52 participants for the single group. Out of 100 questionnaires distributed and collected back, 62 (62%) were considered and included in the analysis, as 28 participants did not complete the assessments, seven dropped out during the initial one-month treatment phase, and three could not be reached for follow-up assessment at three months (Fig. 2).

### Procedure

Participants were scheduled for an individual testing session. Diet clinics adopted a weight loss program for 12 weeks throughout which, participants have a decrease of 500–700 kcal regarding to spontaneous food intake corresponding to a daily decrease of energy intake of 25–45% from baseline needs, a method demonstrated to improve adherence to diet [34]. The dietary pattern used was based on the Mediterranean lifestyle, known for its numerous health benefits [35]; it was diversified and composed of fruits and vegetables, legumes, cereals, and olive oil [36]. The distribution of macronutrient followed the recommendations of the Spanish Society of Community Nutrition: 35% fat (< 10% saturated and 20% monounsaturated), 50% carbohydrates, and 15–20% protein [37].

Additionally, participants were instructed and encouraged to engage in moderate to vigorous-intensity physical activity sessions targeting a maximum heart rate of 65–75% at most for 150 min per week, preferably on most days of the week as per international recommendations [38]. For people who could, a specific weight-lifting training program was advised. Weight lifting is a strength training that helps people gain muscles, which speeds up metabolism and burns more fat on the long term. It is recommended for most people and positively associated with weight-loss strategies and programs.

All physiological and psychological measurements in the questionnaire were administered at baseline and after 12 weeks of dieting. During these assessment sessions, lasting 30–40 min each, patients' height, weight, and body composition were recorded.

The Tanita wall-mounted rod stadiometer, long-trusted for its accuracy and reliability, was used to measure the height [39], and the GAIA Plus device (Jawon Medical, South Korea) recorded weight and body composition, particularly body fat and muscle mass. This device uses the bioelectrical impedance analysis (BIA) simple technique involving the passage of a small electrical current through the body to calculate impedance, which is inversely related to total body water. Thus, a person with lower impedance would have bigger muscles and more body water [40]. BIA measurements should be performed in a standardized manner, ideally at the same time of the day for sequential measurements, to avoid possible variability of results [41].

The three dietitians kept personal records for all clients, which included medical history, family history, and food diary and patterns. The dietitians listened to the desires, needs, and capacities of their clients to set a diet target together and taught them to recognize the feelings of hunger and satiety. The weight and body composition of the participants’ was recorded by the dietitian every 15 days. The weight loss was calculated by subtracting the current weight from the previous recorded weight. Waist circumference was measured using a tape meter.

### Questionnaire

The questionnaire used during the interview was in Arabic, the native language of Lebanon. The first part assessed the sociodemographic information of the participants (age; gender; marital status; educational level divided into primary (less than 5 years of education), complementary (more than 5 years of education), secondary (more than 9 years of education), and university (more than 12 years of education); monthly income divided into no income, low < 1000 USD, intermediate 1000–2000 USD, and high income > 2000 USD), and other variables, such as a family history of eating disorders, BMI, alcohol, tobacco, and caffeine consumption, the perfect and desired weight, and the Total Physical Activity Index (PAI). BMI was calculated by dividing the weight (in kg) by the height in meters squared (m^2^). Alcohol, tobacco, and caffeine consumption were categorized into dichotomous variables (yes/no). The ideal and the desired weight were assessed by two open questions “what is the desired weight that you want to reach” and “what is the perfect weight that you want to reach”. The total PAI was calculated by multiplying the intensity, duration, and frequency of daily activity, reported by participants regarding their physical activity during leisure time [42]. In the original study, the PAI was validated against oxygen consumption (VO2) and heart rate (HR) as variables. Regression analysis revealed a strong positive relationship between the PAI score and VO2 and HR [42].

The second part of the questionnaire consisted of the perception of eating habits among participants. The questions were selected from previous articles [43]. Examples of the asked questions were: “Do you take your weight daily?”, “Do you follow a diet to lose weight?”, “Do you exercise to lose weight?”, “Do you take diet pills to lose weight?”, “Do you take laxatives or vomit to lose weight?”, “Do you starve yourself to lose weight?”, and “Are you under pressure from magazines/TV about losing weight?” and “Do you receive comments from your family concerning weight loss?”. A content validity was done by dietitians and researchers where each items was evaluated for content relevance and representativeness.

The final part of the questionnaire included the following scales:

#### Quality of life short form-12 health survey (SF-12)

The 12-item Short Form Health Survey (SF-12), validated in Lebanon [44], is a Generic Health Rating Scale developed to reproduce the Physical and Mental Component Summary Scores (PCS and MCS, respectively) of a longer survey, the SF-36. Physical and Mental Health Composite Scores (PCS & MCS) are computed using the scores from the twelve questions and range from 0 to 100, where a zero score indicates the lowest level of health and 100 the highest level of health [45]. In this study, Cronbach’s alpha was 0.743.

#### Body dissatisfaction subscale of the eating disorder inventory-second version (EDI-2)

In the present study, body dissatisfaction score was measured using the Eating Disorder Inventory (EDI-2) subscale [46] that assesses the levels of dissatisfaction with the overall body shape and specific body parts. It consists of nine items scored on a 4-point Likert scale from 0 (sometimes, rarely, never) to 3 (always). The total score was calculated by summing the nine items. Higher scores are indicative of greater body dissatisfaction [46]. In this study, Cronbach’s alpha was 0.792.

#### The Rosenberg Self-esteem Scale (RSES)

The RSES is a 10-item scale used to assess beliefs and attitudes towards self-esteem. The psychometric properties of the RSES were evaluated by two studies: the first study examined the psychometric properties of the RSES on college students from eight countries and found adequate to high-reliability results for each country [47]. The second study validated the RSES by translating it into 28 languages and administering it to 16,998 participants across 53 countries. it revealed good psychometric properties across different languages and cultures [48]. The answers were graded on a 4-point Likert scale from 1 (strongly disagree) to 4 (strongly agree). The total score was calculated by summing the ten items [49]. Scores below 15 indicated low self-esteem, and those over 15 indicated higher self-esteem. In this study, Cronbach’s alpha was 0.739.

#### Perceived Stress Scale (PSS)

There are three standard versions of the PSS: the original 14-item form (PSS-14), the PSS-10, and a four-item form. In the original article the PSS-10 demonstrated moderate convergent validity with a good internal consistency (α = 0.78) [50]. In this study, the PSS-10 was used and it was validated in Lebanon [51]. It is a self-report questionnaire used to measure the perception of stress [50]. Ten direct questions scored on a 5-point Likert scale from never (0) to almost always (4) was used to evaluate the levels of experienced stress in the last month [52]. The total score was calculated by summing the ten items, with higher scores indicating higher perceived stress [50]. Scores ranging from 0 to 13 indicate low stress, scores ranging from 14 to 26 indicate moderate stress and scores ranging from 27 to 40 indicate high perceived stress [50]. In this study, the Cronbach alpha was 0.732.

#### Hamilton Anxiety Rating Scale (HAM-A)

The HAM-A, validated in Lebanon [53], is one of the first rating scales to measure the severity of perceived anxiety symptoms. The Arabic version of the HAM-A showed good validity and adequate internal consistency (Cronbach’s α = 0.921) [53]. It consists of 14 symptom-defined elements, identifying both psychological and somatic symptoms. Each item is scored on a basic numeric scoring of 0 (not present) to 4 (severe). The total score, calculated by summing the 14 items, ranged from 0 to 56, with higher scores indicating higher anxiety [54]. In this study, Cronbach’s alpha was 0.894.

#### Hamilton Depression Rating Scale (HAM-D)

The HAM-D, validated in Lebanon [55], was used to measure depression. The HAM-D rating scale includes 21 items, with the last four items not counted toward the total score since these symptoms provide clinical information and are either uncommon or do not reflect depression severity. Therefore, the remaining 17 items of the HAM-D are scored and measure depressive symptoms. The HAM-D is categorized into four categories: No depression (lower than 7), mild depression between 8 – 16, moderate between 17 – 23 and severe equal and higher than 24 [56]. Higher scores would indicate higher depression [57]. In this study, Cronbach’s alpha was 0.729.

### Statistical analysis

IBM® SPSS® Statistics software version 23 (Armonk, New York 10504-1722 United States) was used for data analysis. The mean percentage of missing data was less than 5.0% of the database; therefore, no values were replaced. A descriptive analysis was done using the counts and percentages for categorical variables and mean and standard deviation for continuous measures. The values for skewness and kurtosis were used to prove normal distribution. As the values of the dependent variables were under the acceptable range − 2 and + 2 [58] we have considered that the data normally distributed (the body dissatisfaction subscale: skewness = 0.46, kurtosis = 1.2; physical and mental quality of life: for PCS: skewness = 0.32, kurtosis = − 1.10, MCS: skewness = 0.26, kurtosis = − 0.93). In addition, the normal probability plots of the dependent variables were analyzed and the results showed a normal distribution. The Student’s t-test was used to compare two means whereas the ANOVA test was used when comparison involved three or more groups. Pearson correlation was used for the linear correlation between continuous variables. For categorical variables, the chi-square and Fisher exact tests were used. The paired sample t-test was used to compare continuous variables before and after the diet. Stepwise linear regressions were conducted, taking the body dissatisfaction and the physical and mental quality of life as dependent variables, respectively. We tested for multicollinearity and no similarities had been found between the independent variables. All the variables that showed a *p* < 0.1 in the bivariate analysis were considered important variables to be entered in the model in order to eliminate potentially confounding factors as much as possible [59]. A repeated measures ANOVA was conducted to evaluate factors associated with the change in QOL after the intervention. A value of *p* < 0.05 was considered significant. The internal consistency of the scales was assessed using Cronbach’s alpha.

## Results

Table 1 summarizes the sociodemographic characteristics of the participants. The mean age of the participants was 37.13 ± 11.47 years, with 69.4% females.

**Table 1:** Sociodemographic characteristics of the study sample (N = 62)

	Frequency (%)
*Gender*	
Male	19 (30.6%)
Female	43 (69.4%)
*Marital status*	
Single	27 (43.5%)
Married	33 (53.2%)
Widowed	1 (1.6%)
Divorced	1 (1.6%)
*Education level*	
Primary	1 (1.6%)
Complementary	3 (4.8%)
Secondary	16 (25.8%)
University	42 (67.7%)
*Monthly income*	
No income	9 (14.5%)
< 1000 $	17 (27.4%)
1000–2000 $	25 (40.3%)
> 2000 $	11 (17.7%)
*Smoking*	
Yes	23 (37.1%)
No	39 (62.9%)
*Alcohol*	
Yes	2 (3.2%)
No	60 (96.8%)
*Physical activity during the past 12 months*	
Yes	39 (62.9%)
No	23 (37.1%)
*Family history of eating disorders*	
Yes	25 (40.3%)
No	37 (59.7%)

	Mean ± SD
Age (in years)	37.13 ± 11.47
BMI (kg/m^2^)	28.76 ± 4.91
Body FAT (%)	30.85 ± 12.38
Waist in cm	86.63 ± 31.36
Perfect weight in kg	67.48 ± 10.44
Desired weight in kg	67.70 ± 13.39
Physical Activity Index	36.87 ± 27.31

Table 2 shows participants’ views on eating habits. In the past 30 days, more than half of the participants dieted, exercised, and were under pressure from TV or magazine, and received comments from the family concerning weight loss. The majority did not have any family history of eating disorders (59.7%).

**Table 2:** Dieting behaviors of participants

	Frequency (%)
Dieting to lose weight (past 30 days)	33 (53.2%)
Exercising to lose weight (past 30 days)	33 (53.2%)
Vomiting or taking laxatives to lose weight (past 30 days)	5 (8.1%)
Taking diet pills to lose weight (past 30 days)	10 (16.1%)
Receiving comments from the family concerning losing weight	32 (51.6%)
Pressure from TV, magazine to lose weight	32 (51.6%)

### Comparison of measures before and after diet

Table 3 presents the variation of the measurements of the different scales before and after the diet. Body dissatisfaction, anxiety, waist circumference (in cm), weight (in kg), and body fat percentage were significantly reduced after the diet, compared to before it. Moreover, participants reported a significant increase in the physical and mental quality of life after the diet, compared to before it. No significant variation was found for stress, self-esteem, depression, restrained eating and BMI before and after diet (*p* > 0.05 to all). More than half of the surveyed individuals (54.8%) had no depression before the diet; this percentage increased to 79% afterward. Also, the majority of participants had moderate stress before (96.8%) and after diet (82.3%), and all of them had elevated self-esteem before and after the diet.

**Table 3:** Variation of the measures used before and after diet

	Before diet	After diet	scale ranges	*P* value
	Mean ± SD	Mean ± SD		
Psychological measures				
Body dissatisfaction	15.98 ± 4.99	12.45 ± 4.97	0–27	** < 0.001**
Perceived stress (PSS Scale)	18.59 ± 5.30	17.41 ± 2.03	0–40	0.072
Self-esteem (Rosenberg Scale)	25.27 ± 1.48	25.24 ± 1.00	10–40	0.885
Anxiety (HAMA)	11.23 ± 7.66	3.81 ± 5.39	0–56	**< 0.001**
Depression (HAMD)	7.92 ± 5.47	7.23 ± 2.72	0–52	0.353
Physiological measures				
BMI	28.66 ± 5.01	28.69 ± 5.04	–	0.350
Waist	89.24 ± 31.21	76.76 ± 24.68	–	**0.001**
Weight	79.08 ± 16.83	74.40 ± 15.16	–	** < 0.001**
Body fat	34.21 ± 8.30	30.84 ± 7.27	–	** < 0.001**
Quality of life (SF-12-PCS)	45.04 ± 7.91	49.66 ± 7.68	0–100	** < 0.001**
Quality of life (SF-12-MCS)	44.17 ± 8.47	49.24 ± 7.44	0–100	** < 0.001**

Numbers in bold indicate significant *P* value. The physiological measures were controlled for Physical Activity Index

### Bivariate analysis of factors associated with the body dissatisfaction score before the diet

A significantly higher mean body dissatisfaction score was found in participants with a secondary level of education compared to other groups, receiving comments from their family concerning weight loss, and in those under pressure from TV or magazine, compared to those who did not receive any comments or pressure. A significantly higher mean body dissatisfaction was also associated with a family history of eating disorders and increased BMI, stress score, and anxiety score. However, a higher self-esteem score was significantly associated with decreased body dissatisfaction (Table 4).

**Table 4:** Bivariate analysis of factors associated with the body dissatisfaction score before the diet

	Body dissatisfaction score before the diet	*P* value
	Mean ± SD	
*Gender*		
Male	15.47 ± 4.40	0.597
Female	16.20 ± 5.27	
*Education level*		
Complementary	8.33 ± 7.63	**0.001**
Secondary	18.81 ± 2.85	
University	15.50 ± 4.85	
*Receiving comments from the family concerning losing weight*		
Yes	18.40 ± 4.40	** < 0.001**
No	13.40 ± 4.28	
*Family history of eating disorders*		
Yes	18.20 ± 4.88	**0.004**
No	14.48 ± 4.54	
*Pressure from TV, magazine to lose weight*		
Yes	18.43 ± 3.77	** < 0.001**
No	13.36 ± 4.85	
*Practicing sport during the 12 months*		
Yes	15.23 ± 5.36	0.099
No	17.26 ± 4.09	

	Correlation coefficient	*P* value
Age	0.081	0.535
Body Mass Index	0.593	** < 0.001**
Anxiety	0.449	** < 0.001**
Depression	0.201	0.116
Perceived stress	0.448	** < 0.001**
Self-esteem Scale	− 0.483	**0.002**
Physical Activity Index	− 0.353	**0.028**

Numbers in bold indicate significant *p* values

Variables not included in the table were not significant with body dissatisfaction

### Bivariate analysis of the factors associated with the quality of life before the diet

Lower physical quality of life was significantly associated with higher age, body dissatisfaction, and higher perceived stress. In contrast, a Higher Physical Activity Index was significantly associated with a better physical quality of life. Lower mental quality of life was significantly associated with higher body dissatisfaction, higher perceived stress increased anxiety and depression, whereas male gender, higher self-esteem, and Higher Physical Activity Index were significantly associated with higher mental quality of life (Table 5).

**Table 5:** Bivariate analysis of the factors associated with the quality of life before the diet

	PCS-QOL	*P* value	MCS-QOL	*P* value
	Mean ± SD		Mean ± SD	
*Gender*				
Male	46.86 ± 8.61	0.231	48.27 ± 8.21	**0.010**
Female	44.23 ± 7.54		42.35 ± 8.01	

	Correlation coefficient	*P* value	Correlation coefficient	*P* value
Age	− 0.370	**0.003**	− 0.102	0.432
Body dissatisfaction	− 0.281	**0.027**	− 0.602	** < 0.001**
Perceived stress	− 0.294	**0.020**	− 0.607	** < 0.001**
Self-esteem Scale	0.074	0.570	0.260	**0.041**
HAM-A Score	− 0.244	0.056	− 0.573	** < 0.001**
HAM-D Score	− 0.236	0.065	− 0.331	**0.009**
Physical Activity Index	0.435	**0.006**	0.510	**0.001**

PCS-QOL, physical quality of life; MCS-QOL, mental quality of life

Variables not included in the table were not significant with quality of life

Numbers in bold indicate significant *p* values

### Multivariable analysis of factors associated with body dissatisfaction, physical and mental quality of life before the diet

The results of a first linear regression, taking body dissatisfaction score as the dependent variable, showed that higher BMI (β = 0.44), higher anxiety (β = 0.13) were associated with higher body dissatisfaction scores. However, higher self-esteem (β = − 0.81) and complementary level of education (β = − 4.94) were associated with lower body dissatisfaction (Table 6).

**Table 6:** Multivariable analysis taking the body dissatisfaction score before the diet as the dependent variable

	Unstandardized beta	Standardized beta	*P* value	Confidence interval	
				Lower bound	Upper bound
Body Mass Index	0.440	0.432	** < 0.001**	0.252	0.627
Pressure from TV/magazines to lose your weight	1.833	0.185	0.059	− 0.074	3.740
Anxiety	0.132	0.203	**0.036**	0.009	0.255
Self-esteem	− 0.818	− 0.243	**0.010**	− 1.433	− 0.202
Complementary level of education	− 4.942	− 0.214	**0.020**	− 9.066	− 0.817

Variables entered: Body Mass Index, pressure from TV, magazine to lose your weight, receiving comments from the family concerning losing weight, self-esteem, anxiety, stress, family history of eating disorders and level of education

Numbers in bold indicate significant *p* values

The results of a second linear regression, taking the mental health quality of life as the dependent variable, showed that higher perceived stress (β = − 0.71), higher body dissatisfaction (β = − 0.48), and female gender (β = − 4.09) were associated with lower mental health quality of life, while a Higher Physical Activity Index (β = 0.08) was significantly associated with higher mental health quality of life (Table 7, Model 1).

**Table 7:** Multivariable analysis taking the mental health QOL before the diet as the dependent variable

	Unstandardized beta	Standardized beta	*P* value	Confidence interval	
				Lower bound	Upper bound
*Model 1*					
Perceived stress	− 0.711	− 0.441	0.001	− 1.086	− 0.335
Physical Activity Index	0.086	0.253	0.026	0.011	0.161
Body dissatisfaction	− 0.480	− 0.278	0.024	− 0.892	− 0.068
Female gender compared to males	− 4.094	− 0.211	0.046	− 8.107	− 0.081
*Model 2*					
Physical Activity Index	0.123	0.432	0.007	0.036	0.210

Variables entered in the first model: Body Dissatisfaction Score, perceived stress, anxiety, depression, Physical Activity Index and gender. Variables entered in the second model: age, body dissatisfaction, perceived stress, anxiety, depression and Physical Activity Index

A third linear regression, taking the physical health quality of life as the dependent variable, showed that Higher Physical Activity Index (β = 0.12) was significantly associated with higher physical health QOL (Table 7, Model 2).

### Repeated measures ANOVA

The results of a Repeated measures ANOVA of the factors associated with the change in physical and mental quality of life after the intervention, showed that none of the variables was associated with a change in the physical QOL score (Table 8, Model 1), whereas higher anxiety (β = − 0.45) was significantly associated with a decrease in mental QOL after 3 months (Table 8, Model 2).

**Table 8:** Repeated measures ANOVA of the factors associated with the change in physical and mental quality of life after the intervention

Variable	Beta	*p*	95% CI		Partial eta squared
*Model 1: Physical quality of life score at 3 months*					
Depression	− 0.33	0.101	− 0.73	0.07	0.056
Age	− 0.15	0.095	− 0.32	0.03	0.058
*Model 2: Mental quality of life score at 3 months*					
Anxiety	− 0.45	**0.027**	− 0.86	− 0.05	0.10

When taking the body dissatisfaction as dependent variable, no variable have been found to be associated with it.

## Discussion

To our knowledge, this is the first study in Lebanon that examined the importance of diet and its effects on mental and physical health. It determined how the psychological factors (body dissatisfaction, self-esteem, anxiety, depression and perceived stress) and physiological factors (BMI, waist, weight and body fat) associated with the change in physical and mental quality of life varied before and after the intervention of weight loss.

Our results showed that weight loss diet significantly reduced body dissatisfaction and anxiety and improved physical and mental quality of life. Weight loss interventions programs have psychological consequences and may serve to improve psychological outcome [11]. Weight loss programs allow a person to detect a physical progress, which change positively their mood and improve the body perception, which in turn decrease body image issues, thus increasing the likelihood of weight loss [11]. Also, the strong correlation between anxiety and obesity could be explained by the negative effect of obesity on the patient's self-esteem, and the poor support of the society and the awareness of the body dissatisfaction that could lead to social phobia and an increase of the anxiety level [24, 27, 60]. Indeed studies showed that body dissatisfaction and QOL were associated with higher BMI grades [29, 30]. According to Schwartz and Brownell, obesity can influence body image by causing psychological distress, which has a negative effect on quality of life [20]. Hence, weight loss intervention contribute to a decrease of the body dissatisfaction and an increase of the quality of life. This is in line with previous studies showing that weight loss was associated with decreased body dissatisfaction providing significant physiological benefits and improving both physical and mental health [19–21].

Moreover, the findings of this study showed no significant effect of weight loss programs on perceived stress, self-esteem, and depression, in agreement with other findings [61]. Controversially, some studies found that a change in diet would improve psychological distress [11]. The explanation could be that our study participants had moderate stress and the majority of the sample do not have any depressive symptoms as assessed by the depressive scale. Indeed, an average score for self-esteem scale range was found before and after diet. It is noteworthy that our results were not directly comparable to those of other studies, as the design and the analytic approach used in our study were different. Additionally, the short study duration and the mall sample size did not allow the detection of any significant association. Further studies are needed to clarify these associations.

Moreover, our results showed that BMI was the same before and after the diet. Nevertheless, our results were partly consistent with previous findings [8]. This could be explained by the increase in muscle mass due to physical exercise, which affects the total weight and not BMI.

The results of the multivariable analysis showed that higher anxiety and higher BMI were associated with higher body dissatisfaction. The association between anxiety and BD seems to be reciprocal in literature [25, 26]. Psychological distress might increase once a person had a negative perception of body image [15]. Also, studies have showed that higher BMI was associated with higher body dissatisfaction [29].

This study showed that higher self-esteem and complementary level of education were associated with lower body dissatisfaction. Consistent with previous studies, body dissatisfaction is linked to an impairment in self-esteem. Individuals with high self-esteem are less likely to internalize the sociocultural image of thinness resulting in lower body dissatisfaction [11, 31]. Educational level was not related to body dissatisfaction in a previous study [31] whereas others showed that education can reduce concerns about body image [62].

Three factors were negatively correlated to mental quality of life (QQL): female gender, body dissatisfaction and perceived stress. Several studies showed that female gender was associated with lower mental health QOL [63]. Women could have multiple roles, such as mother, partner, career, worker, and running a household. These factors make women more vulnerable to psychological problems than men, which could affect their body dissatisfaction and their physical and mental quality of life. In the Lebanese society, women are under the pressure of being thin, as a measure of social acceptance, with their QOL being consequently affected by those stressors, depressive factors, and body dissatisfaction. Perceived stress was also negatively correlated with mental health QOL. Chronic stress and negative life events accumulate over time and, therefore, could affect health outcomes and QOL [23].

Higher physical activity was associated with better physical and mental QOL in this study, corroborating previous results [32]. In fact, physical activity increases positive emotions such as interest, excitement, and enthusiasm., enhances positive moods by increasing pleasant, energized feelings, increases muscles strength and the ability to function properly [32, 64].

### Limitations

This study has several limitations. This study was a repeated cross-sectional, so causality cannot be demonstrated and the directional association between factors and BD and QOL cannot be confirmed. The duration of the study was too short to assess any variation in the psychological scales; a longitudinal study would assess better the psychological distress. In addition, selection bias could have occurred since the majority of the respondents were females with high education level and the sample was recruited from three clinics. Also, some scales and the perception of eating habits questions used in this study were not validated in Lebanon. Furthermore, the information collected by the participants could be biased since the study relied on self-report answers. Residual confounding bias is also possible, since there could be factors related to psychological distress variation, body dissatisfaction, and quality of life that were not measured in this study such as previous attempts of weight loss. Accordingly, the results of the study could not be generalized to the population. Future research should consider the use of a larger sample size by examining the change of psychological factors during weight management.

## Conclusion

Overall, this study highlights the importance of a weight loss program and its effects on anthropometric and psychological measures. This study revealed that BMI, anxiety, and low self-esteem were associated with more body dissatisfaction. Perceived stress, body dissatisfaction, and female gender were associated with lower mental health quality of life and physical activity was associated with higher physical well-being.

There is a need to develop intervention programs that could help individuals improving their psychological status throughout a dieting process. In addition, physical exercise in weight loss programs should be included as it plays a positive role in emotional and psychological well-being. Alternatively, a collaborative approach is recommended with mental health/behavioral professionals to treat patients with overweight and obesity. Further interventional studies examining the change of psychological factors during weight management among Lebanese people are warranted.

## Data Availability

The datasets used and/or analyzed during the current study are available from the corresponding author on reasonable request.
